# Synthesis and Characterization of Pure TiO_2_ and TiO_2_‐Doped Bi_2_O_3_ Nanocomposites for Electrochemical Applications

**DOI:** 10.1002/bio.70161

**Published:** 2025-04-02

**Authors:** S. Synthiya, T. Thilagavathi, R. Uthrakumar, Mir Waqas Alam, K. Kaviyarasu

**Affiliations:** ^1^ Department of Physics Government College for Women (Autonomous) Kumbakonam Tamil Nadu India; ^2^ Department of Physics Government Arts College (Autonomous) Salem Tamil Nadu India; ^3^ Department of Physics College of Science King Faisal University Al Hofuf Saudi Arabia; ^4^ UNESCO‐UNISA Africa Chair in Nanosciences/Nanotechnology Laboratories, College of Graduate Studies (CGS) University of South Africa (UNISA) Pretoria South Africa

**Keywords:** low temperature hydrothermal method, malachite green dye, photocatalysis, rhodamine‐B dye, TiO_2_‐doped Bi_2_O_3_ nanocomposites

## Abstract

To remove toxic wastes from water bodies by using a photocatalyst is the objective of this paper. Chemically, pure TiO_2_ and TiO_2_‐doped Bi_2_O_3_ nanoparticles are synthesized using the low temperature hydrothermal method. The properties of the synthesized nanoparticles were studied using various characterization methods. In addition to powder X‐ray diffraction (PXRD) and scanning electron microscopy (SEM), energy‐dispersive X‐ray spectroscopy (EDAX), transmission electron microscopy (TEM), selected area electron diffraction (SAED), and Fourier transform infrared (FTIR) analyses, we studied the structural and surface morphology of the nanoparticles. By analyzing those data, we can determine the average crystallite size, strain, crystallinity, the molecular composition, the grain size of the nanoparticles, the structure, shape, functional groups, and types of bonds between them. Electrochemical impedance spectroscopy (EIS) and cyclic voltammetry (CV) analyses were used for electrochemical studies. Based on these data, a study was conducted on charge transfer and specific capacitance. Photoluminescence (PL) and ultra‐visible absorbance (UV–vis) spectra were used to determine the optical properties. Based on these spectra, it is possible to determine the optical bandgap of the particles and their potential as photocatalysts. Under a visible light radiation source, pure TiO_2_ and TiO_2_‐doped Bi_2_O_3_ nanocomposites were used in the photocatalytic degradation of malachite green (MG) and Rhodamine‐B (RhB) dyes. By studying the observations, we were able to determine the degradation efficiency, as well as the rate constant. Results showed that the TiO_2_‐doped Bi_2_O_3_ nanocomposites showed an increase in degradation efficiency of ~0.8% for RhB dye and ~27.7% for MG dye when compared to pure TiO_2_ nanoparticles.

## Graphical abstract

This work makes use of TiO_2_ nanocomposites and TiO_2_+doped Bi_2_O_3_ nanocomposites to decompose organic dyes. Living organisms may also be negatively affected by dyes that are water soluble as well as contaminating water bodies. The easy solubility of dyes in water makes them ideal for contaminating water bodies. Diverse methods were employed to eliminate these contaminants. The study found that dye degradation with photocatalysis was the most effective method in treating contaminated water using nanocomposites.

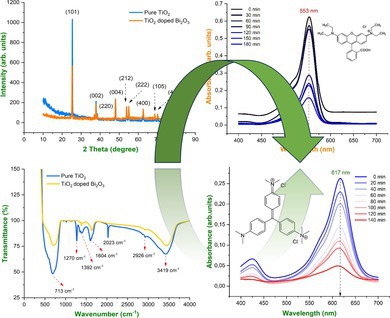

## 1 Introduction

Besides printing, painting, and dyeing agents, rhodamine‐B (RhB) dyes also had an important role in textile, leather, and textile dyeing. As well as being dyes, malachite green (MG) dyes function as antibacterial agents and biological stains and have antifungal properties, making them useful in aquaculture [1]. Although they have a wide range of applications in a wide range of fields, they have significant disadvantages. When consumed, RhB dye causes cancer as it was used in food industries, causing irritation of the skin and eyes, neurotoxicity, and chronic issues. When exposure to MG dye is prolonged, and the concentration level is increased, organisms may suffer severe health issues including mutation, respiratory issues, and damage to organ tissues [2]. Water‐soluble dyes can easily contaminate water bodies and cause several impacts on living organisms. Since dyes are readily soluble in water, they are easily mixed with water bodies and could contaminate water. A variety of methods were used to eliminate these contaminants. Dye degradation with photocatalysis is the most effective method of treating contaminated water. A UV and visible light source was used to stimulate the photocatalysts, resulting in faster degradation of the molecules. A mass water body can benefit from sunlight because it is easy and cost‐effective. Through the use of pure TiO_2_ and TiO_2_ doped Bi_2_O_3_ nanocomposites, we can degrade MG and RhB dyes by photocatalytic degradation.

Since semiconductor nanoparticles [3] possess unique properties when compared with bulk materials, their use in varied industries has been increasing. It is mainly for the purpose of treating pollutants that accumulate in the environment that these nanoparticle applications are used. The purification of water and air was achieved using various semiconductor nanoparticles. As a result, these materials will provide the best photocatalysts and ensure that toxic wastes can be contaminated in all water, air, and soil environments. To determine their photocatalytic activity, semiconductors must be sorted according to their particle size, arrangement, and bandgap energy [4]. In addition to their less toxicity and good photocatalytic activity under visible light, Bi_2_O_3_ nanoparticles are also shown to be very effective as antibacterial agents. The high bandgap of these semiconductors makes them good conductors. At different synthesis temperatures, Bi_2_O_3_ forms six crystal structures: monoclinic (Bi_2_O_3_), tetragonal (Bi_2_O_3_), orthorhombic (Bi_2_O_3_), and triclinic (Bi_2_O_3_). The Bi_2_O_3_ has six crystallographic structures *α*‐Bi_2_O_3_ (monoclinic), *β*‐Bi_2_O_3_ (tetragonal), *δ*‐Bi_2_O_3_ (FCC), γ‐Bi_2_O_3_ (BCC), ε‐Bi_2_O_3_ (orthorhombic), and ῳ‐Bi_2_O_3_ (triclinic) formed at different synthesis temperatures [5, 6].

There has been considerable interest in the application of TiO_2_ nanoparticles [7, 8] for their photocatalytic activities. The high surface‐to‐volume (*s/v*) ratio, their bandgap energy, and their large surface area make them unique. Rutile is the stable phase of TiO_2_ nanoparticles, which exist in three phases. The devices were used to split water, remediate the environment, treat wastewater, and to split sewage. Therefore, TiO_2_ and Bi_2_O_3_ were used as photocatalysts in this study. When light sources are employed, the photocatalysts accelerate contaminant degradation. In the photocatalysts, light absorbs into the bandgap energy, which is then excited, leading to the formation of electron–hole (e^−^/h^+^) pairs on the surface [9, 10]. Consequently, contaminants are removed by the reaction between charge particles and contaminants. A low‐temperature chemical method was used to synthesize nanoparticles, and various characterization techniques were described.

## 2 Experimental Procedure

### 2.1 Synthesis of TiO_2_ and TiO_2_‐Doped Bi_2_O_3_ Nanocomposites

**FIGURE 1:**
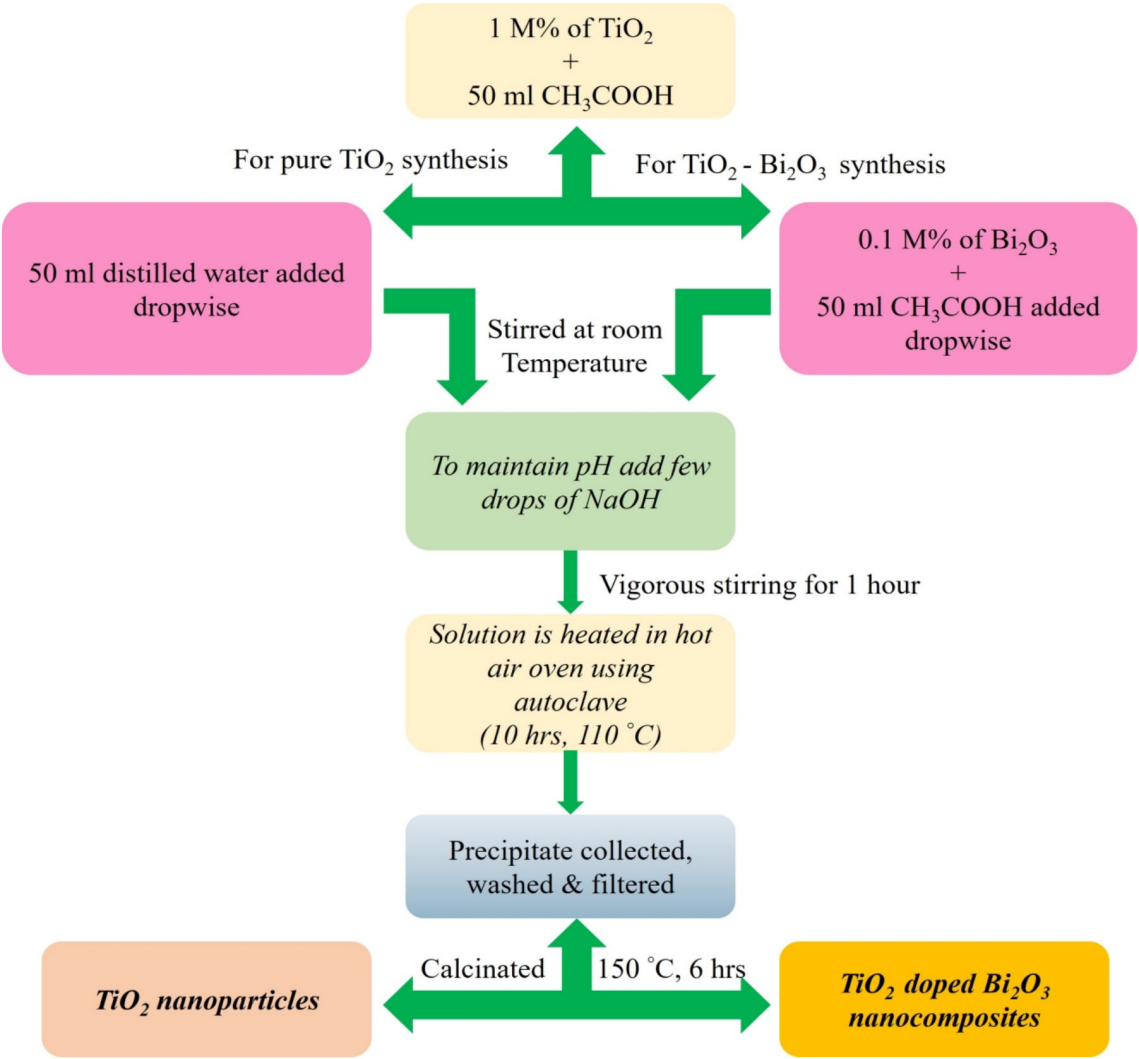
Schematic representation of synthesis for pure TiO_2_ and TiO_2_‐doped Bi_2_O_3_ nanocomposites.

We synthesized TiO_2_ and TiO_2_‐doped Bi_2_O_3_ nanocomposites via the hydrothermal method at low temperatures. As a precursor, 1M% of TiO_2_ sample was diluted with 50 mL of CH_3_COOH in a 500‐mL beaker. In a reactor equipped with a Remi digital high‐speed stirrer, the solution was added at room temperature to a reactor with a high‐speed stirrer. At this point, the solution of 50‐mL of CH_3_COOH containing 0.1 M% of Bi_2_O_3_ was added dropwise to the solution and stirred well for an hour (50 mL of distilled water for pure TiO_2_ nanoparticles) and was added dropwise to the solution and stirred well until the solution became dilute. In order to maintain the pH level of the mixture, sodium hydroxide (NaOH) solution was added. A few hours after stirring the solution, it was added to the hydrothermal autoclave and heated for 10 h at 110°C to precipitate the product. Three times of deionized water and ethanol washing followed by filter paper filtration were used to remove impurities from the precipitate. As a result of this process, the precipitate was dried and calcined for 6 h at 150°C in a hot air oven. With the help of an agate mortar, the powder was crushed into a fine powder. As a result, TiO_2_ nanoparticles are pure, and TiO_2_‐doped Bi_2_O_3_ nanocomposites are obtained. A schematic representation of synthesis for pure and TiO_2_‐doped Bi_2_O_3_ nanocomposites is shown in Figure 1.

### 2.2 Photocatalytic Studies

An equilibrated stock solution of dyes (RhB and MG) containing 10 mg/L was prepared. Taking 250 mL of dye solution and adding 20 mg of photocatalyst (pure TiO_2_ and TiO_2_‐doped Bi_2_O_3_ nanocomposites) to a 500‐mL beaker was the procedure. In order to achieve the adsorption–desorption equilibrium, the solution was stirred in the dark for 20 min before exposure to light. According to the measurement, the maximum absorption wavelengths for both TiO_2_ and TiO_2_‐doped Bi_2_O_3_ nanocomposites were 553 and 554 nm of RhB dye and 617 nm of MG dye. For the study of dye degradation by photocatalysis, the solutions were exposed to sunlight. Using the UV–vis spectrophotometer model UV‐1900i with a wavelength range of 190–1100 nm, the degradation of dyes was calculated for photocatalytic studies.

### 2.3 Characterization Techniques

The PXRD studies were carried out by powder X‐ray diffractometer, model‐X'Pert Pro‐PAnalytic with 15 KVA UPS support (CuK_α_ irradiation, λ = 1.5406 Å) at 40 kV and 30 mA. The FESEM and EDAX images were obtained from the scanning electron microscope with electron dispersive X‐ray spectrometer model—Quanta FEG 250 with magnification 30X–300 kX and resolution of 30 kV at low and high vacuum conditions (3.0 and 1.2 nm). The TEM and SAED studies were done using Tecnai G^2^ 20 S‐TWIN TEM with resolution of 0.14 nm and magnification range 25X – 1030 kX. The FTIR studies were done by Fourier transform infrared spectrometer model—Spectrum Two by Perkin Elmer with range 4000–400 cm^−1^. The electrochemical studies were studied using cyclic voltameter/impedance analyzer model—Versa STAT MC by Princeton Applied Research with operating frequency range 1 Hz – 1 MHz. The PL studies were done by Varian Cary Eclipse photoluminescence spectrophotometer. UV–vis data were studied by UV–vis spectrophotometer model—Lambda 35 by Perkin Elmer with operating range 190–1100 nm. For photocatalytic studies, the degradation of dyes was calculated using UV–vis spectrophotometer model—UV‐1900i with operating range 190–1100 nm. With a scanning electron microscopy and a Quanta FEG 250 Electron Dispersive Spectrometer model, powder X‐ray diffractometers were used to conduct the PXRD studies with magnification 30X–300 kX and resolution 30 kV in low and high vacuum conditions at 3.0 and 1.2 nm, respectively. A Tecnai G2 20 S‐TWIN TEM with a resolution of 0.14 nm and magnification range of 25X–1030 kX was used for the TEM and SAED studies. FTIR analysis was conducted using a Fourier Transform Infrared Spectrometer, Spectrum Two, manufactured by Perkin Elmer and capable of measuring 4000–400 cm^−1^ of wavelength. A Versa STAT MC Cyclic Voltameter was used to conduct electrochemical investigations, with a frequency range of 1 Hz to 1 MHz. Varian Cary Eclipse photoluminescence spectrophotometers were used for PL studies. Using a Perkin Elmer UV–vis spectrophotometer model Lambda 35 with 190–1100 nm operating range, UV–vis data were examined.

## 3 Results and Discussion

### 3.1 Powder X‐Ray Diffraction and Surface Morphology Pattern

**FIGURE 2:**
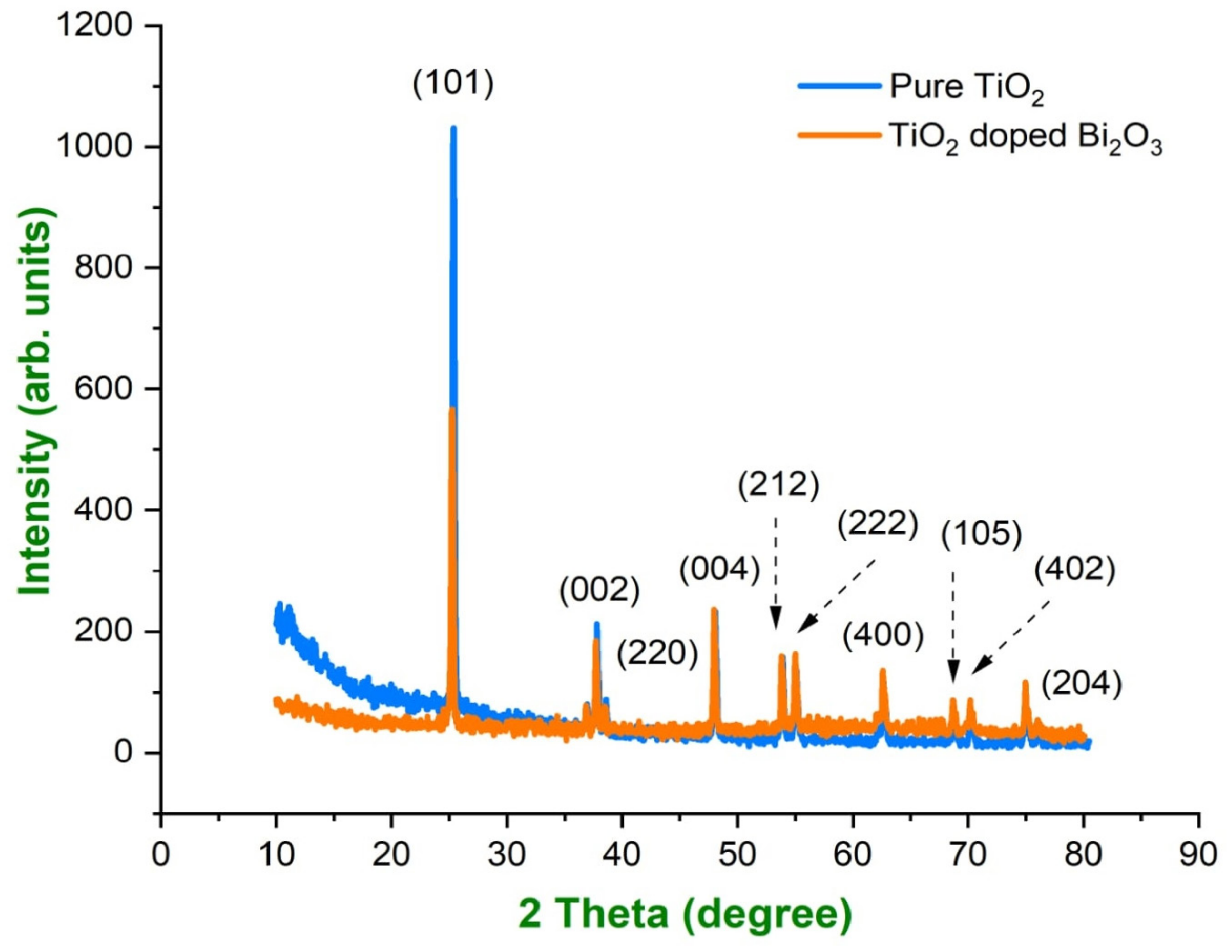
XRD pattern of pure TiO_2_ and TiO_2_‐doped Bi_2_O_3_ nanocomposites.

Analyzing the surface and structure of a material using this method is one of the most powerful analytical techniques. Based on the sharp peaks in XRD, we can conclude that the materials are crystalline. As shown in Figure 2, the XRD patterns of TiO_2_ nanoparticles and TiO_2_‐doped Bi_2_O_3_ nanocomposites were also obtained. An XRD analysis can determine the phase, orientations, crystallinity lattice parameters, defects, crystallite size, and other properties like micro strain and dislocation density. TiO_2_ nanoparticles were clearly in the anatase phase, as shown in Figure 2, with high intensity peak positions at positions 25.26°, 53.90°, and 70.38°, respectively, with corresponding peaks at positions (105) and (204) [11, 12, 13]. TiO_2_‐doped Bi_2_O_3_ nanocomposites were shown to have displacement and addition of peaks due to Bi_2_O_3_ addition. In the XRD pattern of TiO_2_‐doped Bi_2_O_3_ nanocomposites, additional peaks can be seen at (002), (212), and (222), which confirm the presence of Bi_2_O_3_ nanoparticles. In the XRD data, there was no evidence of impurities, except for Bi_2_O_3_ and TiO_2_. The average crystallite size of the nanoparticles was calculated using the Debye Scherrer formula (1).

(1) $$D=\frac{\mathrm{k\lambda}}{\beta \cos\theta }$$

where “*D*” is the crystallite size, “*λ*” is the wavelength of the X‐rays, which is 1.5405 Å, “*k*” is the particle factor = 0.94, “*β*” is the full width half maximum, and “*θ*” is the Bragg's diffraction angle. The dislocation density (*δ*), and micro strains (*ε*) were found by the following equations (Equations 2 and 3):

(2) $$\delta =\frac{1}{D^{2}}\;\left(\text{lines}\;m^{-2}\right)$$

(3) $$\varepsilon =\frac{\beta }{4\sin\theta }$$

We performed the least square line regression using Equation (4) to determine the average crystallite size of the nanoparticles using the modified Debye Scherrer method.

(4) $$\ln\;\beta =\ln\;\frac{1}{\cos\theta }+\ln\;\frac{\mathrm{k\lambda}}{D}$$

With the help of the full width half maximum and total broadening, the Williamson–Hall (W‐H) method was used to determine the average crystallite size and micro strain of the nanoparticles. It can be expressed as Equation (5), where “*T*” is the total broadening and “*ε*” is the micro strain the nanoparticles undergo. It can be expressed using Equation (5), in which *β*_*T*_ is the total broadening, followed by a micro strain of the nanoparticle.

(5) $$\beta_{T}\;\cos\theta =\mathrm{k\lambda D}+4\varepsilon \;\sin\theta$$

**FIGURE 3:**
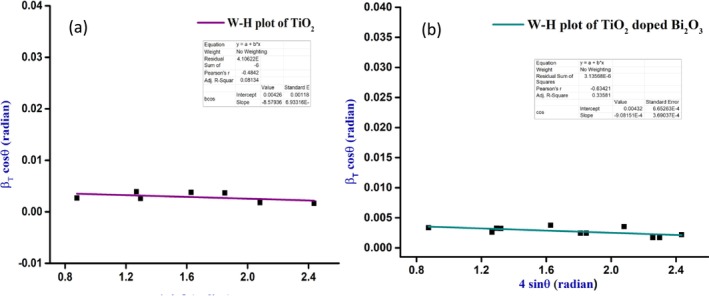
(a, b) W‐H plot for pure TiO_2_ and TiO_2_‐doped Bi_2_O_3_ nanocomposites.

TiO_2_‐doped Bi_2_O_3_ nanocomposites had an average crystallite size of 54 nm, while TiO_2_ nanoparticles had an average crystallite size of 70.68 nm as shown in Table 1. The dislocation density and micro strain were also calculated from the method, and the values obtained were 0.4646 × 10^−3^ and 0.4166 × 10^−3^ nm^−2^ for the dislocation density and 1.992 × 10^−3^ and 1.8116 × 10^−3^ for the strain of pure and TiO_2_‐doped Bi_2_O_3_ nanocomposites, respectively. The average crystallite size determined from the modified equations was 36 and 27 nm, respectively. Thus, the strain and grain size for pure TiO_2_ and TiO_2_‐doped Bi_2_O_3_ nanoparticles obtained from the W‐H plot were −8.57936 × 10^−4^ and 32 nm and 6.65263 × 10^−4^ and 32 nm, respectively, as shown in Figure 3a,b. For both the W‐H plot and the modified Debye–Scherrer method, the crystallite size was nearly the same. Positive and negative strain values were caused by lattice expansion and compression. Using the TiO_2_‐doped Bi_2_O_3_ nanoparticles as an example, the crystallinity was calculated, and the value was 22 nm for pure TiO_2_ nanoparticles and 70 nm for TiO_2_‐doped Bi_2_O_3_ nanoparticles, indicating that crystallinity increased during doping.

**TABLE 1:** Average crystallite size calculated from various methods and crystallinity of pure TiO_2_ and TiO_2_‐doped Bi_2_O_3_ nanocomposites.

Sample	Average crystallite size (nm)			Crystallinity
	Debye Scherrer method	Modified Debye Scherrer method	W‐H method	
Pure TiO_2_	54.125	36	32.55	22.48
TiO_2_‐doped Bi_2_O_3_	54.27	37.42	32.095	70.68

### 3.2 Scanning Electron Microscopy and EDAX Spectroscopy

**FIGURE 4:**
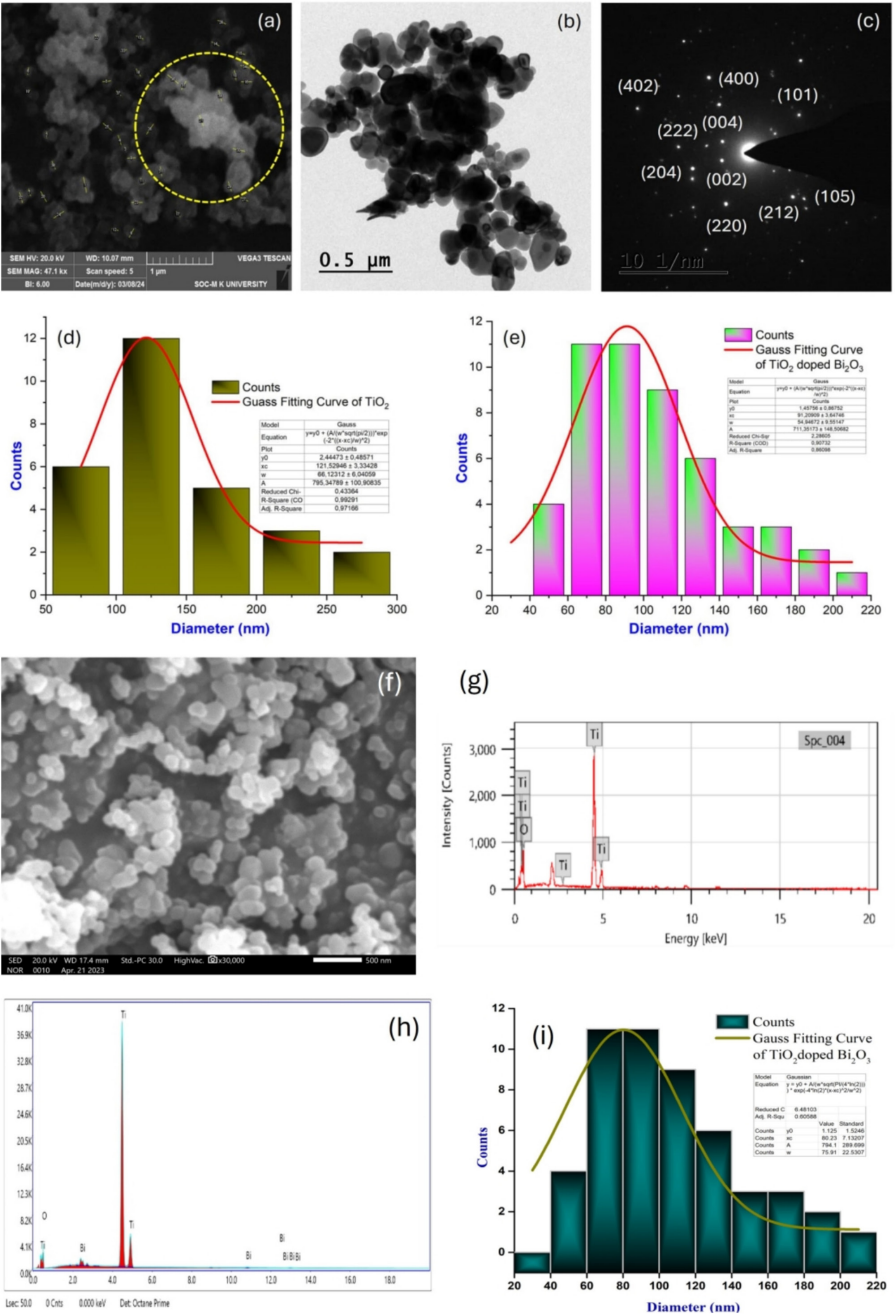
(a) SEM, (b) TEM, (c) SAED, and (h) EDAX image of TiO_2_‐doped Bi_2_O_3_ nanoparticle; (d and e) particle distribution of TiO_2_ and TiO_2_‐doped Bi_2_O_3_ nanoparticles using SEM image; (f) SEM and (g) EDS image of TiO_2_ nanoparticle; (i) particle distribution image using TEM image.

A scanning electron microscope (SEM) was used to measure the nanoparticles' structure, shape, and grain size. The surfaces of the elements were captured with a high magnification camera. At a magnification of 500 nm, Figure 4a illustrates SEM images of pure TiO_2_ nanoparticles and TiO_2_‐doped Bi_2_O_3_ nanoparticles. With the agglomeration of irregular spherical nanoclusters, the porous structure emerged. A high capacitance was achieved by using doped nanoparticles that were porous compared with pure nanoparticles. In addition to enhancing photocatalytic performance, the composite material will have a higher porosity. According to ImageJ software, the average grain size of doped nanoparticles was 129 nm, and the average grain size of pure nanoparticles was 146 nm. When doping element was added to the nanomaterial, a reduction in particle size was observed. As shown in Figure 4d–f, the particle size distribution of nanoparticles on the surface was calculated by Gauss fit. Nanoparticles made from TiO_2_ and nanocomposite with TiO_2_‐doped Bi_2_O_3_ nanoparticles had average particle sizes of 121 and 130 nm, respectively. Particle sizes were nearly identical when calculated using ImageJ software and Gauss fitting. An effective analytical technique for determining elemental compositions of materials is energy‐dispersive X‐ray spectroscopy (EDS). This result is based on the results of the EDS analysis of the sample; as shown in Figure 4g,h, they are both pure nanoparticles, with no contamination or impurity. The EDAX spectrum confirms that different elements are present and in what proportions. A figure shows the atomic compositions of the synthesized nanomaterials as shown in the following paragraph. Oxygen (O) and titanium (Ti) are the only two peaks visible in Figure 4g. Oxygen makes up ~69.79% and titanium make up 30.21%. In Figure 4h, the peaks for the TiO_2_ doped Bi_2_O_3_ nanoparticles can be seen. In these compounds, 69.16% of O, 30.52% of Ti, and 0.32% of Bi make up the atomic percentages of Ti, Bi, and O. Bi's low atomic percentage is a result of a small amount of bismuth oxide being added during synthesis.

### 3.3 Transmission Electron Microscopy and SAED Pattern

From high‐resolution transmission electron microscope images, we calculated surface morphology, nanomaterial nature, and average grain size. As shown in Figure 4b, a TiO_2_‐doped Bi_2_O_3_ nanoparticle was imaged using TEM. The nanoparticles had an interconnected nanoparticle morphology and were spherical in shape [14, 15]. Using ImageJ software, 103 nm was calculated as the average crystalline size of the nanoparticles. According to Figure 4i, the nanoparticle grain size was 80 nm, and the distribution of grain size was determined by gauss fit. In Figure 4c, TiO_2_‐doped Bi_2_O_3_ nanoparticles were depicted with SAED patterns. As a result of the presence of discrete rings on the bright spot, the particles were confirmed as nanocrystalline. As seen in the XRD data, the SAED pattern and XRD pattern had the same *d‐*spacing and had spots and rings that corresponded to the planes (402), (204), (222), (002), (212), (222), (105), and (101) of the XRD patterns. As a result, we can confirm there are no other elements present in the nanoparticles, except for TiO_2_ and Bi_2_O_3_. The Bi_2_O_3_ agglomerations had an average diameter of 81 nm, were evenly distributed on the surface of the nanoparticles, and covered most of the nanoparticles' circumferences. A decrease in particle size of about 81 nm was observed in the samples. In their cross‐sectional morphologies, the produced samples exhibited an adhesive texture, indicating that TiO_2_‐doped Bi_2_O_3_ nanoparticles were distributed evenly. Bi_2_O_3_ appears to have been deposited on the top surface of spherical nanoclusters.

### 3.4 FTIR Spectroscopy

**FIGURE 5:**
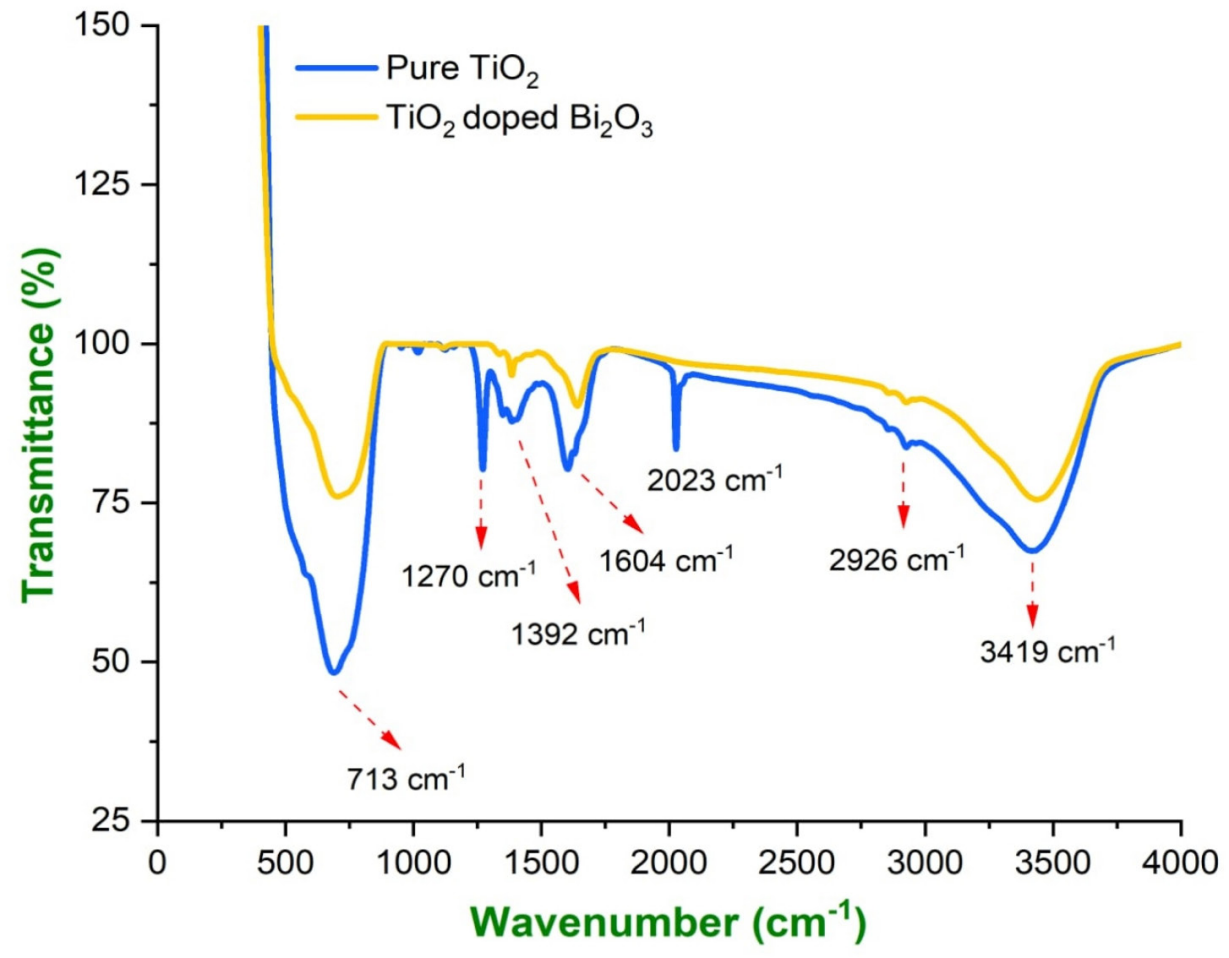
FTIR spectrum of pure TiO_2_ and TiO_2_‐doped Bi_2_O_3_ nanoparticles.

As a non‐destructive technique, the FTIR spectroscopy can identify unknown elements, determine chemical bonds of nanomaterials, and determine their functional groups. As shown in Figure 5, the FTIR spectra of TiO_2_ and TiO_2_‐doped Bi_2_O_3_ nanoparticles range from 4000 to 400 cm^−1^. TiO_2_‐doped Bi_2_O_3_ nanoparticles exhibit strong absorption peaks at 713 cm^−1^ owing to their presence of metal oxygen vibrations and at 689 cm^−1^ for pure TiO_2_ due to their strong stretching vibrations between Ti‐O‐Ti bonds. It is believed that the sharp peaks between 1300 and 1400 cm^−1^ were due to the Ti‐O‐Ti vibrational mode. Approximately 3417–3000 cm^−1^ are vibration bands that stretch hydroxyls. An illustration of the stretching vibration of the C‐H bond can be found at 3000 cm^−1^. The bands located at 2963, 2907, and 2809 cm^−1^ correspond to a stretching vibration of CH, CH_2_, and CH_3_. A correlation exists between the stretching vibration of the aromatic ring's C=C bond and the vibration at 1600 cm^−1^. The bending vibrations of CH, CH_2_, and CH_3_ can be assigned to the bands in the 1500–1400 cm^−1^ range, while the vibrations of the methoxy group O‐CH_3_ and alcohol group C‐OH are responsible for the bands in the 1300–1200 cm^−1^ range. Water molecules adsorb on nanoparticle surfaces causing bending vibration at 1604 cm^−1^ corresponding to the sharp absorption peak observed at this wavelength. A 2926 cm^−1^ spectrum was observed for C‐H aliphatics with stretch vibrations. As shown in Table 2, it indicates the types of bonds and vibrational modes that are present in nanoparticles based on their wavenumbers. There were broad bands observed at 3300–3600 cm^−1^, and stretching vibrations were observed due to the presence of hydroxyl groups [16, 17, 18]. FTIR spectra indicate that Ti and Bi elements are present in nanoparticles, based on the sharp absorption peaks. Nanoparticles synthesized from organic compounds are thus confirmed to be of high purity.

**TABLE 2:** The absorption peaks and their corresponding functional groups and mode.

Absorption peak wavenumber	Functional group bond type and mode
713 cm^−1^	Bi‐O, metal–oxygen vibration
689.73 cm^−1^	Ti‐O‐Ti, stretching vibration mode
1392 cm^−1^	Ti‐O‐Ti, vibrational mode
1604 cm^−1^	O‐H bending vibration of water molecules, adsorbed on the surface
2926 cm^−1^	C‐H aliphatic compound, stretch vibration
3419 cm^−1^	OH‐O group and water molecules adsorbed on the surface, stretching vibration

### 3.5 Electrochemical Analysis

#### 3.5.1 Cyclic Voltammetry and Electron Impedance Spectroscopy

**FIGURE 6:**
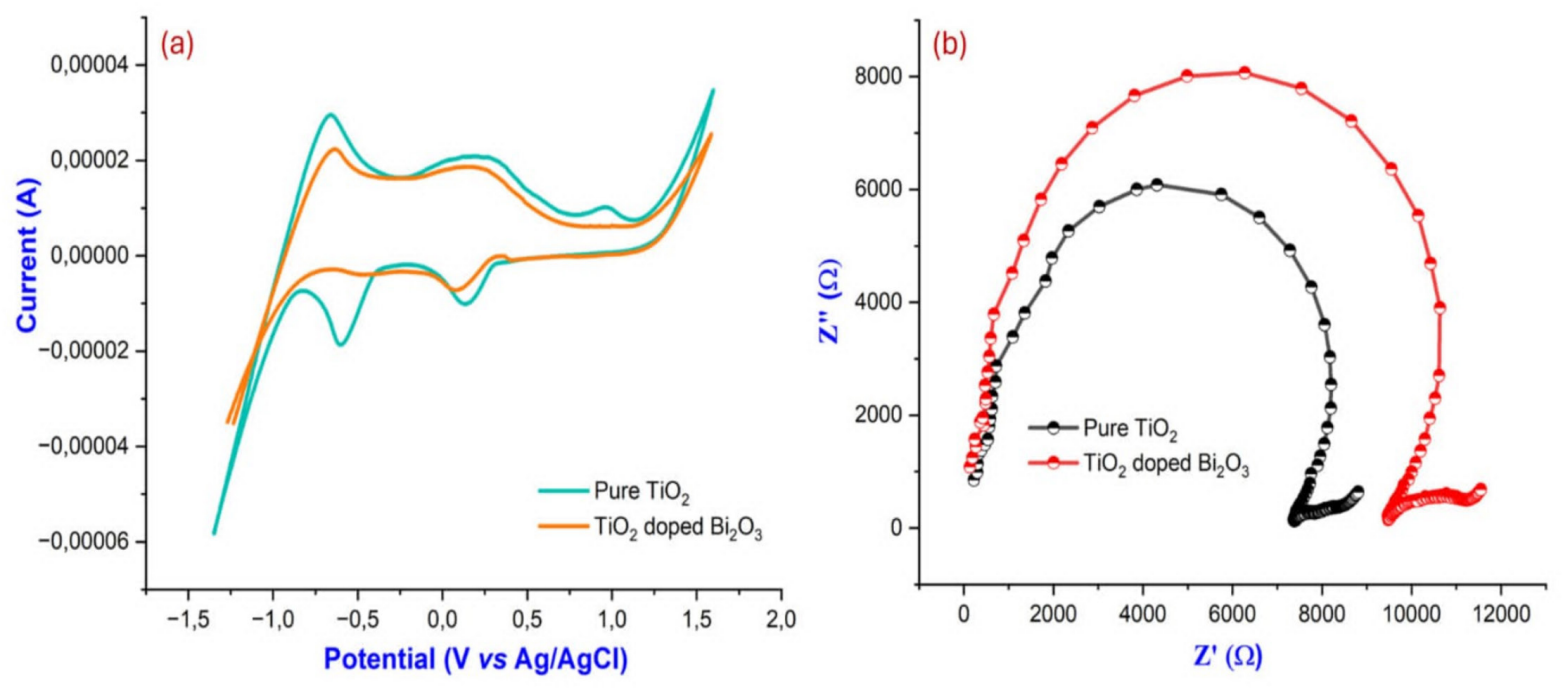
(a) CV curve and (b) EIS spectra of pure TiO_2_ and TiO_2_‐doped Bi_2_O_3_ nanocomposites.

A CV curve was used to study the electrochemical behavior of pure TiO_2_ and TiO_2_‐doped Bi_2_O_3_ nanoparticles in the potential range of −2 to +2 V and current range of −60 to 40 μA at the scan rate of 10 mV/s. Data from CV were analyzed in order to obtain several qualitative insights. Pure TiO_2_ and TiO_2_‐doped Bi_2_O_3_ nanoparticle CV curves are shown in Figure 6a. Calculation of the specific capacitance of synthesized materials was performed using Equation (6), where

(6) $$C=\frac{I\Delta T}{m\Delta V}$$

where “*C*” is the specific capacitance in F g^−1^, “*I*” is the galvanostatic discharge current in Ampere, discharge time in second, “*V*” is the voltage range, and “*m*” is the mass of the active material in gram. This equation can be rewritten as $C=\frac{A}{2\text{mkΔv}}$, where “*A*” is the area under the CV curve, “*m*” is the mass of the material, *ΔV* is the voltage window, and scan rate was given by “*k*.” Pure TiO_2_ and TiO_2_‐doped Bi_2_O_3_ nanoparticles had specific capacitances of 464 and 330 F/g, respectively. These properties enable pure TiO_2_ nanoparticles and TiO_2_‐doped Bi_2_O_3_ nanocomposites to be used as supercapacitors in energy storage devices. As ions penetrate an electrode material for a longer period of time in an electrolyte that has a low scan rate, the specific capacitance is increased [19, 20]. To study the recombination of charge and capacitance of the materials, EIS spectra were obtained as Nyquist plots. Using semicircular Nyquist plots, you can identify the mechanism of charge transfer, while using straight Nyquist plots, you can determine whether the material tested is a capacitor [21, 22]. Applied frequency ranges of 100 kHz–25 Hz were studied for EIS spectra. After fitting the Nyquist plot, the semicircle can be seen in Figure 6b. Charge transfer resistance (RCT) between counter electrodes and redox electrolytes can be seen at the higher frequency end of the semicircle. A comparison of RCT values for pure TiO_2_ nanoparticles with TiO_2_‐doped Bi_2_O_3_ nanoparticles can be seen in Figure 6b. Based on the diameter of the curve, the RCT value was calculated. TiO_2_ (TiO_2_‐doped Bi_2_O_3_ nanocomposites) recombination takes place at intermediate frequencies at the TiO_2_/electrolyte interface at the middle of the semiconductor. The electrolyte ion diffusion was observed in the low frequency region. With increasing semicircle diameter, RCT value decreases, which causes recombination rate to decrease and resistance to increase [14, 23]. In comparison with TiO_2_‐doped Bi_2_O_3_ nanocomposites, TiO_2_ nanoparticles have a low RCT value, which results in high electron–hole transport. According to CV studies, TiO_2_‐doped Bi_2_O_3_ nanocomposites have a low specific capacitance when compared with pure nanoparticles as the specific capacitance decreases with the increase in RCT.

### 3.6 Photoluminescence Spectroscopy

**FIGURE 7:**
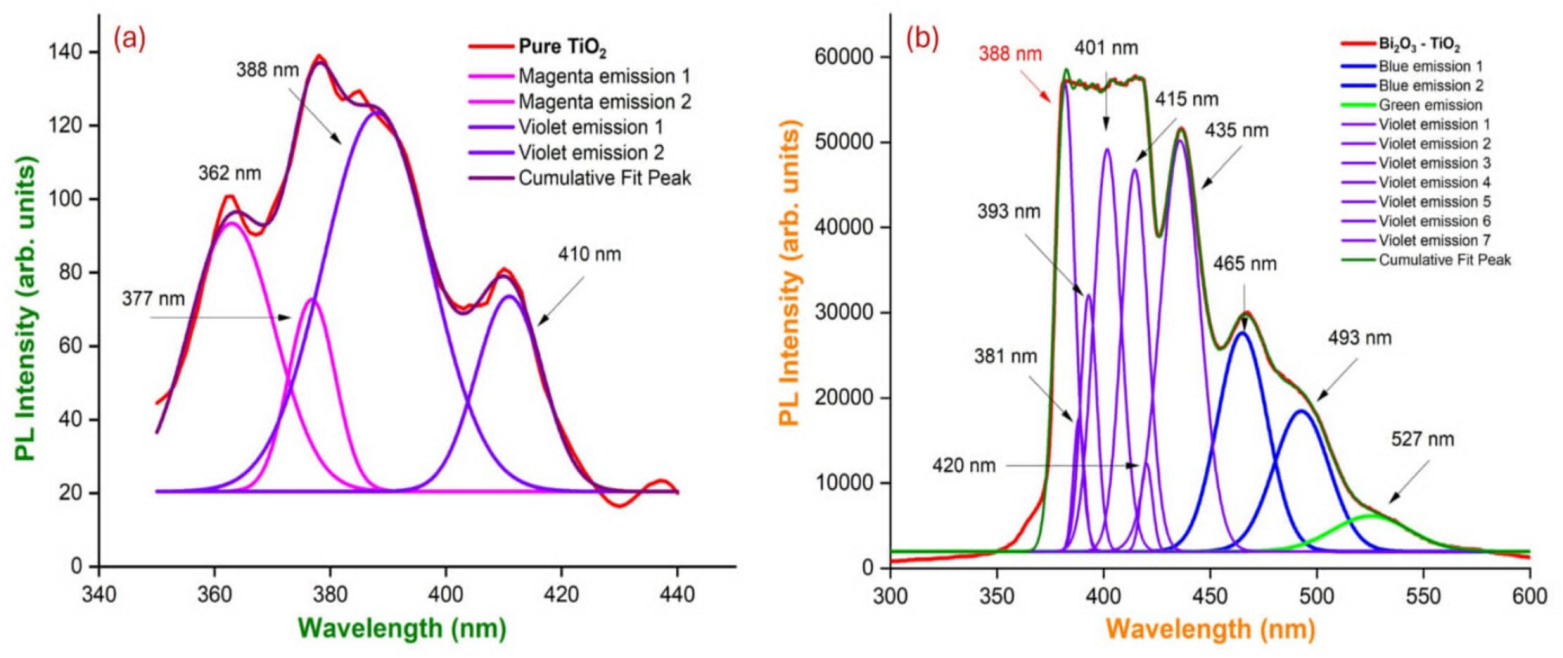
(a) Deconvolution of PL spectrum of pure TiO_2_ and (b) TiO_2_‐doped Bi_2_O_3_ nanocomposites.

PL spectra can be used to analyze the electronic structure of materials without damaging them. For both pure TiO_2_ and TiO_2_‐doped Bi_2_O_3_ nanocomposites, deconvolution of PL spectra [20, 24] was performed, and emission wavelengths were determined by fitting Pl data with Gaussian functions. In addition to broad peaks, there were various emission peaks observed. A pair of electrons and holes were formed by the nanoparticle's absorption of light, which caused the emission. As shown in Table 3, it shows the wavelength of the light produced by the nanoparticles. In the spectra, we see that TiO_2_ nanoparticles emit radiation around 362 and 377 nm, hence their imperceptibility. In their wavelength ranges between 410 and 438 nm, they emit violet light. As a result of the interaction between Bi_2_O_3_ and TiO_2_, violet emission is observed at wavelengths 388, 381, 393, 401, 415, 420, and 435 nm, blue emission at wavelengths 462 and 493 nm, and green emission at 527 nm for nanoparticles TiO_2_‐doped Bi_2_O_3_. The excitation wavelengths of pure TiO_2_ and TiO_2_‐doped Bi_2_O_3_ nanocomposites were used to calculate the electronic bandgap energies as shown in Figure 7a,b, which were ~3.28 and ~3.24 eV, respectively. A phase of anatase was present in the pure TiO_2_ nanoparticles. Doped nanoparticles will exhibit a greater photocatalytic activity because their bandgap energy is slightly decreased.

**TABLE 3:** Emission of light observed for the given wavelength from the PL data.

Sample	Optical bandgap energy E_g_ (eV)	Emission wavelength λ emi (nm)	Colour of emission
TiO_2_	~3.28 eV	362, 377	Magenta
		388, 410	Violet
TiO_2_ doped Bi_2_O_3_	~3.24 eV	388, 381, 393, 401, 415, 420, 435	Violet
		462, 493	Blue
		527	Green

### 3.7 UV–Vis Spectroscopy

**FIGURE 8:**
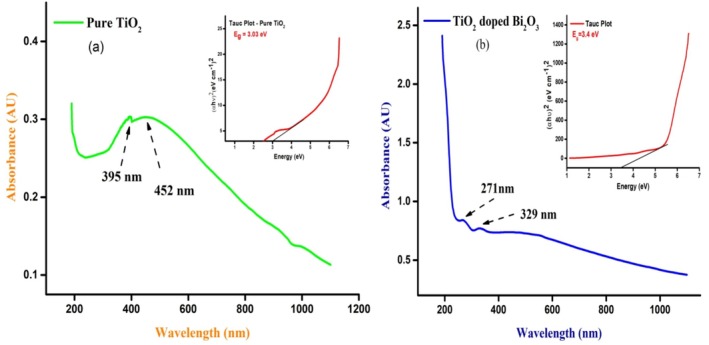
(a, b) UV–vis spectra and *Tauc plot* of pure TiO_2_ and TiO_2_‐doped Bi_2_O_3_ nanocomposites.

An optical property of the nanoparticles can be studied with UV–vis spectroscopy to find a suitable photocatalyst. In addition to the optical bandgap, the concentration, shape, and size of the materials were determined from UV–vis measurements. In order to determine the optical bandgap energy of the material, we used the *Tauc plot*. We will determine which photocatalyst will be most suitable for the photocatalytic reaction. An ultraviolet–visible spectrum of pure TiO_2_ and TiO_2_‐doped Bi_2_O_3_ nanocomposites and a *Tauc plot* of these nanoparticles are shown in Figure 8a,b. In the case of TiO_2_ nanoparticles, the peak at 395 nm was formed by the nanoparticles, while in the case of Bi_2_O_3_, the peak was formed by the nanoparticles. As shown in Equation (7), the Tauc equation [21, 25] is the following:

(7) $$\mathrm{\alpha h\nu}=A\;{\left(\mathrm{h\nu}–E_{g}\right)}^{n}$$

where “α” is absorption coefficient, “h” is plank constant, “ν” is frequency, “A” is the energy dependent constant, E_g_ is the optical bandgap energy of the material, and “n” is the nature of transition (n = ½, 3/2, 2, 3); here, *n* = 1/2 for direct transition. In comparison to TiO_2_, TiO_2_‐doped Bi_2_O_3_ nanoparticles have bandgap energies of ~3.03 and ~3.4 eV, respectively, according to a *Tauc plot*. The confinement of electrons will result in a decrease in the band gap energy of the molecule with increasing particle size. As semiconductors, the synthesized nanoparticles were used in the study.

### 3.8 Photocatalytic Reaction

**FIGURE 9:**
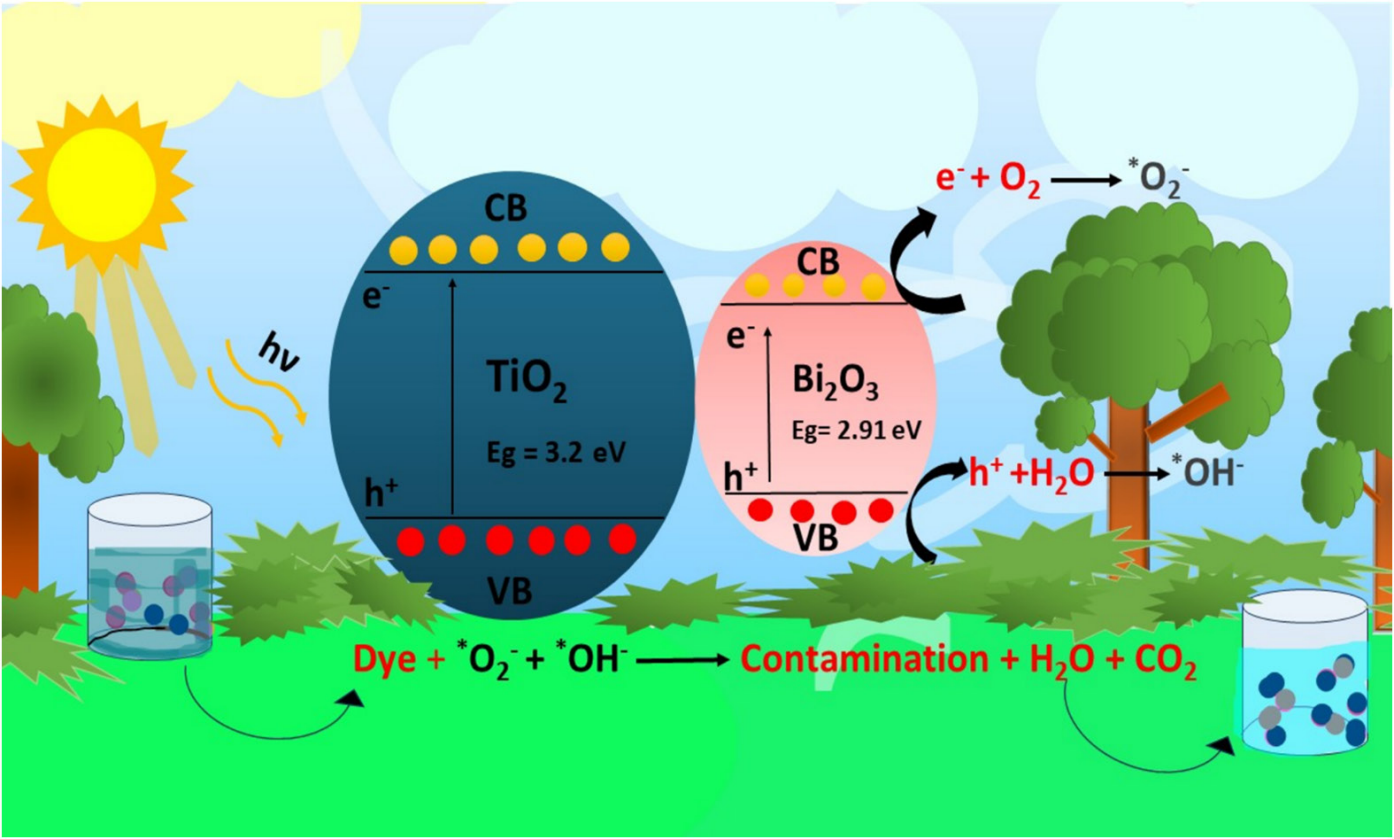
Schematic diagram representing the photocatalytic mechanism.

In a wide range of fields, photocatalysts were used to achieve a variety of objectives. To remove hazardous wastes from waterbodies, we are seeking to synthesize the most effective photocatalyst. Using both pure TiO_2_ and TiO_2_‐doped Bi_2_O_3_ nanocomposites under visible light, the photocatalytic degradation of malachite green dye and Rhodamine‐B dye was studied. A photocatalytic analysis was conducted on the synthesized nanoparticles. The degradation efficiencies, rate constants, and photocatalytic behaviors of the nanoparticles were calculated. Figure 9 illustrates the mechanism of photocatalysis [18, 21]. As the contaminated water was placed under light, the photocatalyst was added to it. The electron–hole pair is generated after radiation is absorbed and moves to the surface of the photocatalyst, where redox reactions occur to degrade the photocatalyst. A first‐order kinetics is followed by the photodegradation process.

#### 3.8.1 Preparatory Methods for Photocatalytic Degradation

**FIGURE 10:**
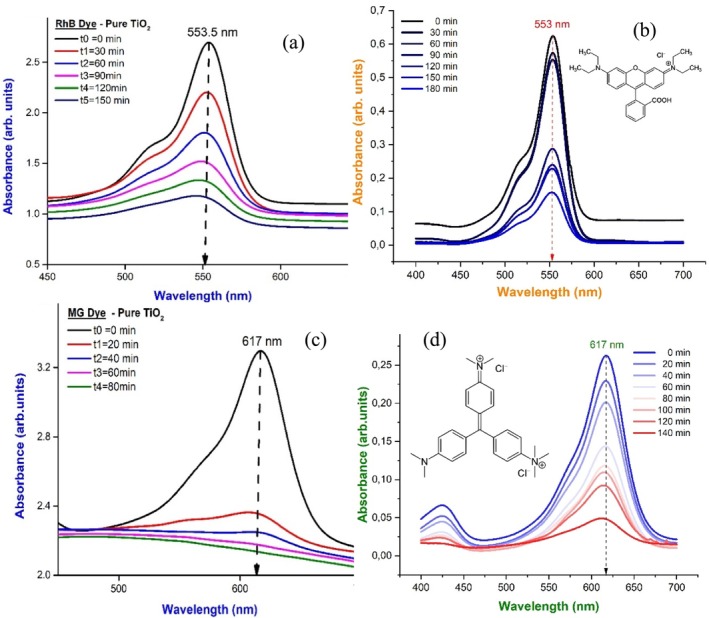
The UV–vis spectrum of RhB dye degradation at different time intervals by (a) pure TiO_2_ and (b) TiO_2_‐doped Bi_2_O_3_ nanocomposites and the UV–vis spectrum of MG Dye degradation at different intervals by (c) pure TiO_2_ and (d) TiO_2_‐doped Bi_2_O_3_ nanocomposites.

As part of preparing the stock solution, 10 mg/L RhB dye (MG) dye was added along with 20‐mg catalysts (pure and TiO_2_‐doped Bi_2_O_3_ nanocomposites). To study the photocatalytic degradation of dye, 250 mL of the stock solution was poured into a 500‐mL beaker, and the catalyst was added. The samples were irradiated in sunlight for about 150 min (80 min for MG) for pure TiO_2_ and 180 min (140 min for MG) for TiO_2_‐doped Bi_2_O_3_, and the percentage of degradation of dye was calculated for every 30 min (20 min for MG) using a UV–vis spectrophotometer. In Figure 10a,b, RhB dye degradation is shown by (a) pure nanoparticles and (b) TiO_2_‐doped Bi_2_O_3_ nanocomposites for the given time interval. For RhB dye degradation, TiO_2_ and TiO_2_‐doped Bi_2_O_3_ nanocomposites had maximum absorption values of 553 and 554 nm, respectively, while MG dyes, as shown in Figure 10c,d, had a maximum absorption value of 617 nm. The photocatalysts used were semiconductor nanoparticles with bandgap energies between 1.5 and 3.5 eV.

From the relation, it is possible to calculate the rate constant of photocatalytic activity, $\ln\left(\frac{A_{0}}{A_{t}}\right)=\mathrm{kt}$ where absorption value determined from UV–vis spectra. As Beer–Lambert's law states that absorption is proportional to concentration, we can determine the rate constant from the corresponding absorption value [14, 26].

(8) $$\ln\left(\frac{C_{0}}{C_{t}}\right)=\mathrm{kt}-\ln\left(C₀/\mathrm{Cₜ}\right)=\mathrm{kt}$$

**FIGURE 11:**
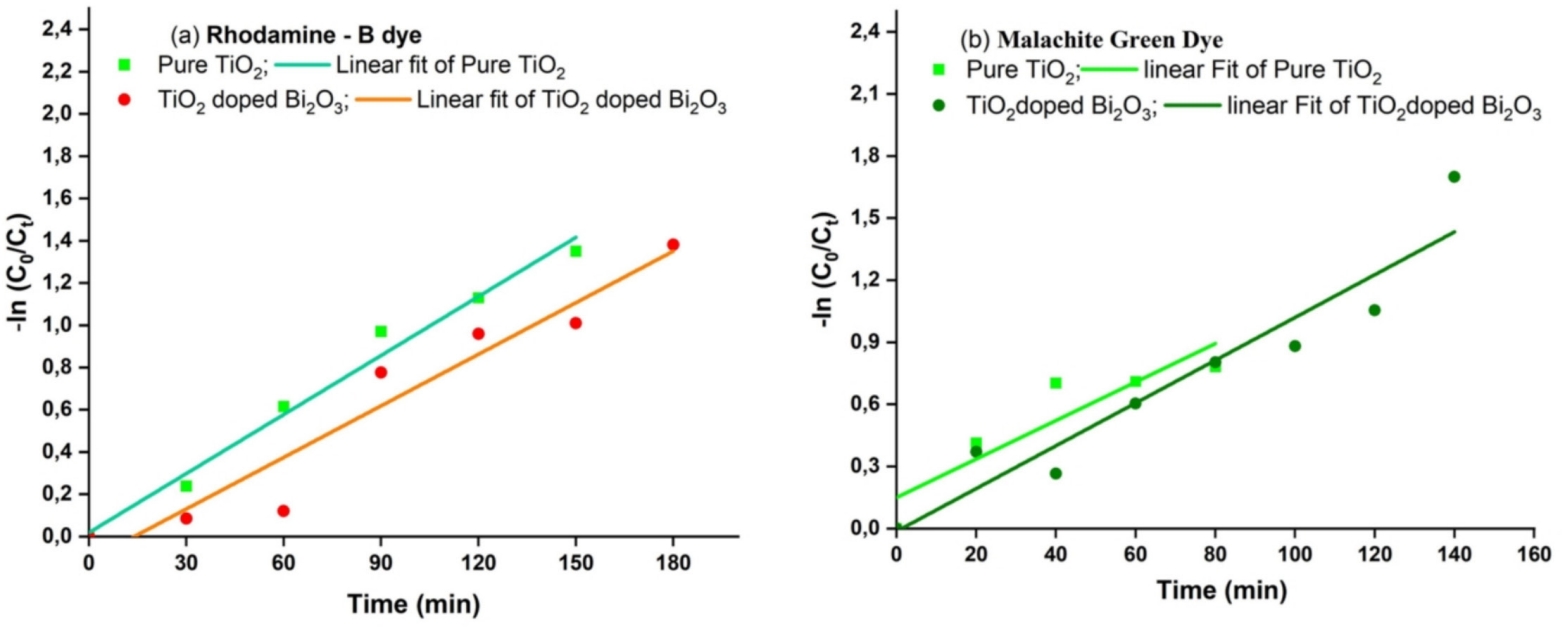
The apparent rate constant of (a) RhB dye and (b) MG dye using pure and TiO_2_ doped Bi_2_O_3_ nanocomposites.

Figure 11a,b displays the slope of the −ln(*C*₀/*C*ₜ) versus time plot, which is used to calculate the degradation rate constant (*k*). By analyzing the linearity of the graph, we can infer that the photodegradation of the dyes under sunlight follows pseudo‐first‐order kinetics. This is because, in a pseudo‐first‐order reaction, the plot of −ln(C₀/Cₜ) versus time typically yields a straight line, indicating a constant rate of degradation. The degradation rate constants for the RhB dye were found to be 0.00931 min^−1^ for pure TiO₂ and 0.08134 min^−1^ for TiO₂ doped with Bi₂O₃. Similarly, for the MG dye, the rate constants were 0.00928 min^−1^ for pure TiO₂ and 0.010335 min^−1^ for TiO₂ doped with Bi₂O₃. This approach assumes that the photodegradation process follows a nonlinear pattern, which aligns with the observed linearity of the −ln(C₀/Cₜ) plot. Therefore, the nonlinear model, represented by the first‐order rate law, is appropriate for describing the dye degradation process, confirming that the reaction rate is proportional to the concentration of the dye at any given time.

In Equation (9), dye degradation is given as a percentage efficiency by Equation (9):

(9) $$\%D=\frac{A_{0}-\mathrm{At}}{A_{0}}\times 100$$

**FIGURE 12:**
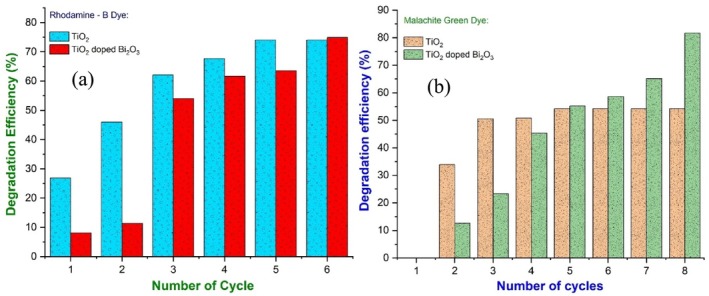
Degradation efficiency percentage of (a) RhB dye and (b) MG dye using pure TiO_2_ and TiO_2_‐doped Bi_2_O_3_ nanocomposites.

The initial and final absorbance values were A_o_ and A_t_, respectively. In Figure 12a,b, as a result of different cycles of 30 and 20 min each, the degradation efficiency (%) of RhB and MG dye is shown for TiO_2_ nanoparticles and TiO_2_‐doped Bi_2_O_3_ nanocomposites, respectively. TiO_2_ and TiO_2_‐doped Bi_2_O_3_ nanocomposites showed ~74.8% and ~74.8% degradation efficiency for degrading RhB dye, respectively, and MG dye showed ~54% and ~81.7% degradation efficiency. In addition to increasing photocatalytic activity, dopants also enhanced their activity.

## 4 Conclusion

By using the low temperature hydrothermal method, the pure TiO_2_ and TiO_2_‐doped Bi_2_O_3_ nanocomposites were synthesized. In addition to comparing the XRD data with Debye Scherrer, modified Debye Scherrer, and W‐H methods, the XRD data show that the synthesized nanoparticles were in the anatase phase, and their average crystallite size was determined. Lattice compression and lattice expansion cause negative and positive strains in nanoparticles. Based on the EDS spectra, it was confirmed that the pure TiO_2_ nanoparticles contained only Ti and O, while the TiO_2_‐doped Bi_2_O_3_ nanoparticles contained only Bi, Ti, and O. As a result of the presence of metal‐oxygen bonds (Bi, Ti), sharp absorption peaks were observed in the FTIR spectra. Utilizing ImageJ software and origin software, we calculated the particle size distribution curve with a Gauss fit based on the SEM and TEM data. Nanoparticles were confirmed polycrystalline by the SAED pattern. A CV study was performed to determine the use of the nanoparticles for supercapacitors. Based on the EIS spectra, it was concluded that pure TiO_2_ nanoparticles have a high electron–hole transfer due to their low RCT value compared to doped nanoparticles. Using Tauc plots, PL and UV–vis spectra of TiO_2_ nanoparticles yield optical bandgaps of 3.28 and 3.03 eV, respectively, while those of TiO_2_ doped Bi_2_O_3_ nanocomposites yield optical bandgaps of 3.24 and 3.4 eV. By doing so, the particles were ensured to be in the anatase phase and semiconductor in nature. We investigated degradation efficiencies in sunlight (visible region) using photocatalytic methods. MG dye degradation efficiency is ~81.7% for TiO_2_‐doped Bi_2_O_3_ nanoparticles, while RhB dye degradation efficiency is ~74% for TiO_2_ nanoparticles and ~54% for TiO_2_‐doped Bi_2_O_3_ nanocomposites. As a result, we conclude that the doping agent enhances the photocatalytic activity.

## Author Contributions

**S. Synthiya:** conceptualization, investigation, writing – original draft, formal analysis, funding acquisition, validation, methodology, resources. **T. Thilagavathi:** conceptualization, methodology, software, funding acquisition, project administration, data curation, supervision, writing – review and editing, visualization, writing – original draft, formal analysis. **R. Uthrakumar:** validation, methodology, writing – review and editing, formal analysis, supervision, software, data curation, project administration, investigation, visualization. **Mir Waqas Alam:** investigation, validation, visualization, funding acquisition, formal analysis, writing – review and editing, resources, data curation. **K. Kaviyarasu:** conceptualization, funding acquisition, writing – review and editing, visualization, validation, resources, project administration, supervision, software.

## Ethics Statement

The authors have nothing to report.

## Conflicts of Interest

The authors declare no conflicts of interest.

## Data Availability

The datasets generated during and/or analyzed during the current study are available from the corresponding author upon reasonable request. All data generated or analyzed during this study are included in this published article.
